# Evaluating ChatGPT-4’s Diagnostic Accuracy: Impact of Visual Data Integration

**DOI:** 10.2196/55627

**Published:** 2024-04-09

**Authors:** Takanobu Hirosawa, Yukinori Harada, Kazuki Tokumasu, Takahiro Ito, Tomoharu Suzuki, Taro Shimizu

**Affiliations:** 1 Department of Diagnostic and Generalist Medicine Dokkyo Medical University Shimotsuga Japan; 2 Department of General Medicine Okayama University Graduate School of Medicine, Dentistry and Pharmaceutical Sciences Okayama Japan; 3 Satsuki Home Clinic Tochigi Japan; 4 Department of Hospital Medicine Urasoe General Hospital Okinawa Japan

**Keywords:** artificial intelligence, large language model, LLM, LLMs, language model, language models, ChatGPT, GPT, ChatGPT-4V, ChatGPT-4 Vision, clinical decision support, natural language processing, decision support, NLP, diagnostic excellence, diagnosis, diagnoses, diagnose, diagnostic, diagnostics, image, images, imaging

## Abstract

**Background:**

In the evolving field of health care, multimodal generative artificial intelligence (AI) systems, such as ChatGPT-4 with vision (ChatGPT-4V), represent a significant advancement, as they integrate visual data with text data. This integration has the potential to revolutionize clinical diagnostics by offering more comprehensive analysis capabilities. However, the impact on diagnostic accuracy of using image data to augment ChatGPT-4 remains unclear.

**Objective:**

This study aims to assess the impact of adding image data on ChatGPT-4’s diagnostic accuracy and provide insights into how image data integration can enhance the accuracy of multimodal AI in medical diagnostics. Specifically, this study endeavored to compare the diagnostic accuracy between ChatGPT-4V, which processed both text and image data, and its counterpart, ChatGPT-4, which only uses text data.

**Methods:**

We identified a total of 557 case reports published in the *American Journal of Case Reports* from January 2022 to March 2023. After excluding cases that were nondiagnostic, pediatric, and lacking image data, we included 363 case descriptions with their final diagnoses and associated images. We compared the diagnostic accuracy of ChatGPT-4V and ChatGPT-4 without vision based on their ability to include the final diagnoses within differential diagnosis lists. Two independent physicians evaluated their accuracy, with a third resolving any discrepancies, ensuring a rigorous and objective analysis.

**Results:**

The integration of image data into ChatGPT-4V did not significantly enhance diagnostic accuracy, showing that final diagnoses were included in the top 10 differential diagnosis lists at a rate of 85.1% (n=309), comparable to the rate of 87.9% (n=319) for the text-only version (*P*=.33). Notably, ChatGPT-4V’s performance in correctly identifying the top diagnosis was inferior, at 44.4% (n=161), compared with 55.9% (n=203) for the text-only version (*P*=.002, *χ*^2^ test). Additionally, ChatGPT-4’s self-reports showed that image data accounted for 30% of the weight in developing the differential diagnosis lists in more than half of cases.

**Conclusions:**

Our findings reveal that currently, ChatGPT-4V predominantly relies on textual data, limiting its ability to fully use the diagnostic potential of visual information. This study underscores the need for further development of multimodal generative AI systems to effectively integrate and use clinical image data. Enhancing the diagnostic performance of such AI systems through improved multimodal data integration could significantly benefit patient care by providing more accurate and comprehensive diagnostic insights. Future research should focus on overcoming these limitations, paving the way for the practical application of advanced AI in medicine.

## Introduction

### Diagnostic Excellence

Diagnostic excellence involves accurately and efficiently diagnosing a wide range of conditions [1]. Achieving this requires a multifaceted approach [2], including effective collaboration among medical professionals, patients, families, and clinical decision support systems (CDSSs). Each plays a pivotal role, as follows: medical professionals bring their expertise and judgment, patients and families provide essential health information and context, and CDSSs offer data-driven insights, enhancing the collective decision-making process.

### CDSSs for Diagnostic Excellence

CDSSs are computer-based tools that assist medical professionals in a wide range of clinical decisions, including diagnosis, treatment planning, medication ordering, preventive care, and patient education [3]. Research has shown that CDSS interventions significantly improve diagnostic accuracy [4], a key aspect of diagnostic excellence [5]. For instance, interventions involving a CDSS in the diagnosis of common chronic diseases demonstrated significant improvements [6]. Accurate diagnosis entails more than identifying a disease; it involves understanding the patient’s unique health context, ensuring timely and appropriate treatment, reducing misdiagnosis risk, and ultimately improving patient outcomes [7]. In the rapidly evolving health care environment, maintaining high standards of diagnostic precision becomes increasingly crucial.

### Artificial Intelligence in Medicine

CDSSs are broadly categorized into 2 types [3]: knowledge-based systems, which are grounded in medical guidelines and expert knowledge; and non–knowledge-based systems, using artificial intelligence (AI) or statistical pattern recognition for clinical data analysis.

The integration of AI into clinical settings is advancing rapidly. AI systems in medicine range from assisting in diagnostic imaging and analysis to optimizing patient treatment plans [8,9]. These systems are being increasingly adopted in hospitals and clinics [10], significantly contributing to enhanced diagnostic accuracy and efficiency.

However, the integration of AI into clinical settings brings transformative potential but also faces several hurdles. Challenges include ensuring data privacy [11], addressing the lack of large and diverse training data sets, and maintaining the interpretability of AI-generated recommendations to align with ethical standards [12,13]. Real-world obstacles, such as resistance from health care professionals due to trust issues in AI’s diagnostic suggestions, underscore the complexity of AI integration into clinical practice.

### Advancements in Large Language Models

A notable advancement in AI is the use of large language models (LLMs). As a subset of non–knowledge-based systems, LLMs are specialized forms of generative AI systems that process and generate human-like text based on extensive textual data training [14]. They are adept at tasks like translation, summarization, and even creative writing. In clinical practice, generative AI systems using LLMs have shown promise in summarizing patient history, integrating medical records, analyzing complex data streams, and enhancing communication between patients and medical professionals [15,16], demonstrating their utility in handling complex medical language and concepts. Such advancements not only improve the efficiency of medical documentation but also offer novel approaches to generating differential diagnoses, showcasing the innovative application of LLMs in clinical settings.

### Multimodal Artificial Intelligence in Diagnostics

Integrating multimodal data, including text and images, presents technical challenges. Successful integration in other fields, such as autonomous driving technologies that combine multisensory observation data to navigate [17], offers a potential model for health care. Recent developments in generative AI systems, including Google Gemini (previous Google Bard [18]) and ChatGPT-4 with vision (ChatGPT-4V), have enabled the processing of both text and image data. This integration is essential for providing a comprehensive clinical overview. Although effectively combining data from different data sources remains a challenge, the development of multimodal AI models that incorporate data across modalities enabled broad applications that include personalized medicine and digital health [19]. For example, a multimodal model developed from the combination of images and health records could classify pulmonary embolism [20]. Another multimodal model could differentiate between common respiratory failure [21]. Among publicly available generative AI systems, the ChatGPT series, particularly ChatGPT-4V, developed by OpenAI and released in September 2023, stands out [22,23]. It accepts both text and image data [24,25], demonstrating impressive performance in various applications.

Preliminary studies in various fields, including medicine [26-28] and others [29-31] have shown the effectiveness of ChatGPT-4V. Some of these studies have highlighted its efficacy in interpreting medical images [26,28], though they were limited in scope. However, clinical image data includes a wide range of elements, from physical examinations to various investigation results. The full impact of image data integration on diagnostic accuracy is yet to be thoroughly explored.

### Study Objectives

This study directly addressed the gaps identified in the current understanding of multimodal AI’s application in clinical diagnostics. By comparing the diagnostic accuracy of ChatGPT-4V and without vision across detailed case reports, and examining the impact of image data integration, we aimed to provide concrete evidence on the value and challenges of incorporating generative AI into clinical flows. Our objectives were shaped by the need to better understand how multimodal AI can be optimized to support diagnostic excellence, ultimately contributing to the advancement of medical diagnostics through technology.

## Methods

### Overview

**Figure 1:**
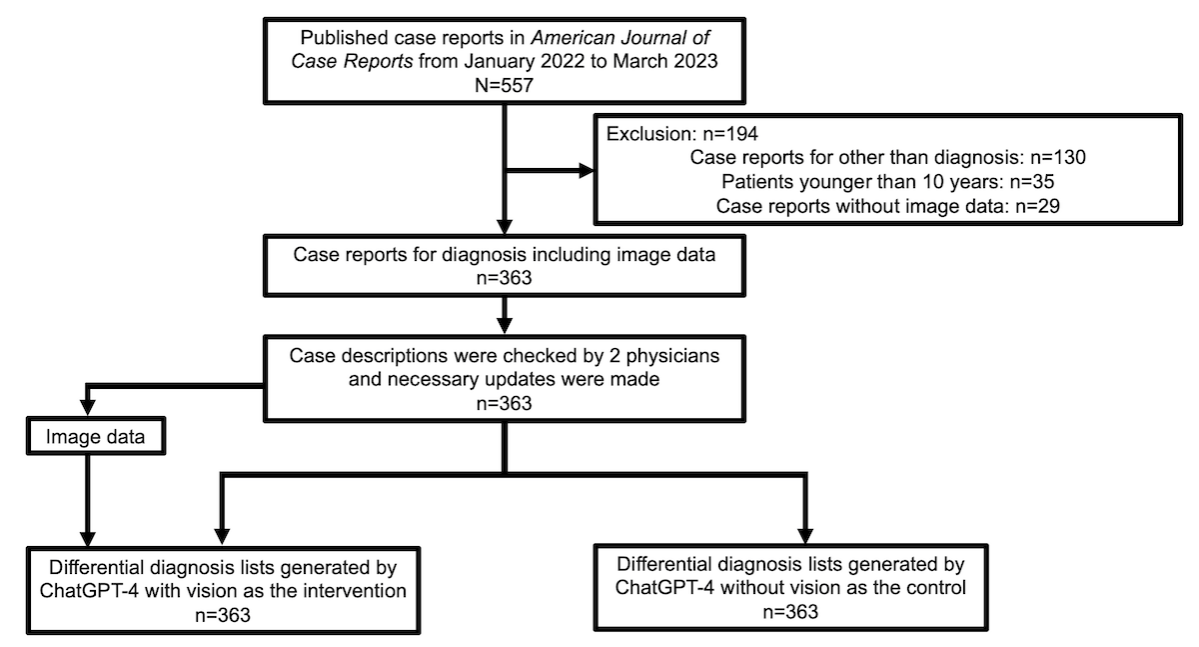
Study design.

We conducted an experimental study to assess the diagnostic accuracy of multimodal generative AI systems using data from a large number of case reports. This study was conducted in the Department of Diagnostic and Generalist Medicine (General Internal Medicine) at Dokkyo Medical University. This study involved several steps: preparing the data set and control, preparing image data, generating differential diagnosis lists by ChatGPT-4V, and evaluating the diagnostic accuracy of these differential diagnosis lists. A flow chart of the study’s methodology is presented in Figure 1.

### Ethical Considerations

This study used published case reports, and thus ethical committee approval was not applicable.

### Preparing Data Set and Control

We used the data set from our previous study (T Hirosawa, Y Harada, K Mizuta, T Sakamoto, K Tokomasu, T Shimizu, unpublished data, November 2023). The data set comprised case descriptions and final diagnoses, sourced from the *American Journal of Case Reports*, spanning January 2022 to March 2023. This peer-reviewed journal covers diagnostically challenging case reports from various medical fields. A total of 557 case reports were identified. The exclusion criteria were carefully chosen based on previous studies for CDSSs [32] and ChatGPT-4V [28] to ensure the focus remained on diagnostically challenging adult cases with relevant image data. Specifically, cases were excluded for the following reasons: nondiagnosis (130 cases), patients younger than 10 years (35 cases), and the absence of image data (29 cases). The included case reports were refined into case descriptions by the primary researcher (TH) and double-checked by another researcher (YH). From the included case reports, we extracted a case description until the final diagnosis was made in the “case report” section. We removed sentences that directly assessed the diagnosis to minimize bias in generating differential diagnoses. This step ensures that the differential diagnoses generated by ChatGPT-4 are based solely on the unbiased clinical presentation of the case. After brush-up, we formatted these case descriptions for input into ChatGPT-4. A typical case description included demographic information, chief complaints, history of present illness, results of physical examinations, and investigative findings leading to diagnoses. The final diagnoses were typically determined by the authors of the case reports. For example, in a case report titled “Levofloxacin-Associated Bullous Pemphigoid in a Hemodialysis Patient After Kidney Transplant Failure” [33] we extracted from “A 27-year-old female with hemodialysis was admitted for evaluation of a worsening bullous rash and shortness of breath over the last 3 days...” to “...Although the swab PCR test for VZV and HSV was negative, there was still concern about disseminated herpes zoster, as the patient was immunosuppressed” as a case description. Additionally, the final diagnosis was levofloxacin-associated bullous pemphigoid.

In the next step, we used ChatGPT-4 without vision to develop the top 10 differential diagnosis lists based on the data of case descriptions. Two expert physicians independently evaluated whether the final diagnosis was included in the lists, and any discrepancies were resolved through discussion. Therefore, the differential diagnosis lists and data of physicians’ evaluation of the lists from a total of 363 case reports were included as the control in this study.

### Preparing Image Data

All figures and tables of included case descriptions were standardized to a resolution of 96 dots per inch in JPEG format to balance detail with file size, facilitating efficient processing by ChatGPT-4V without compromising the quality necessary for accurate diagnostic inference. When multiple figures or tables were present in a case description, they were compiled into a single JPEG file, each annotated with a file number in the upper-left corner. If image data exceeded the upload size limit, the images were resized to half their original size while preserving image quality, using the Preview application (version 11.0; Apple Inc) on a Mac computer.

### Generating Differential Diagnosis Lists by ChatGPT-4V

We used ChatGPT-4V, a multimodal generative AI system developed by OpenAI, from October 30, 2023, to November 9, 2023. Additional training or reinforcement for diagnosis was not performed. The prompt was constructed as follows: “Identify the top 10 suspected illnesses based on the attached files with file names indicated in the left upper corner of each image, and the provided case description. List these illnesses using only their names, without providing any reasoning AND describe the proportion of the case description and the provided files to develop your suspected illness list (case description + all files = 100%): (copy and paste the case descriptions).” This design was intended to explicitly guide ChatGPT-4V to not only generate a list of possible diagnoses but also reflect on how each type of data influenced its conclusions, providing insights into the AI’s diagnostics process. Apart from the prompt and file names, the text data input to ChatGPT-4V remained the same as the control, ChatGPT-4 without vision. The first generated list was used as the differential diagnosis list. The chat history was cleared before entering each new case description. Moreover, the data control settings for chat history were disabled. The details of ChatGPT-4V and ChatGPT-4 without vision are shown in Table 1.

**Table 1:** The details of ChatGPT-4 with vision and ChatGPT-4 without vision in this study.

Details	ChatGPT-4 with vision (intervention) [24]	ChatGPT-4 without vision (control) [22]
Short name	ChatGPT-4V	ChatGPT-4
Prompt	Identify the top 10 suspected illnesses based on the attached files with file names indicated in the left upper corner of each image, and the provided case description. List these illnesses using only their names, without providing any reasoning AND describe the proportion of the case description and the provided files to develop your suspected illness list (case description + all files =100%): (copy and paste the case descriptions)	Tell me the top 10 suspected illnesses for the following case: (copy and paste the case descriptions)
Text input	Same case descriptions with the above prompt and referred file number	Same case descriptions with the above prompt
Image input	Image data in JPEG format with a resolution of 96 dots per inch	No image data
Output	The top 10 differential diagnosis lists and the proportion of weight between text data and image data contributing to development of the differential diagnosis list	The top 10 differential diagnosis lists
Evaluations	By 2 independent physicians; any discrepancies were resolved by another physician	By 2 independent physicians; any discrepancies were resolved by another physician
Release date	September 2023	March 2023
Access date	From October 30, 2023, to November 9, 2023	From June 22, 2023, to June 29, 2023
Data control for chat history	Off	Off

### Evaluation for Differential Diagnosis Lists by Physicians

Two expert physicians, TI and T Suzuki, independently evaluated whether the final diagnoses were included in the differential diagnosis lists. The evaluation was binary, with 1 indicating inclusion and 0 indicating exclusion. A score of 1 indicated that the differential closely matched the final diagnoses. This close match was defined not merely by the presence of the correct diagnosis within the list but by the relevance and clinical appropriateness of the differentials in relation to the final diagnosis. A score of 1 indicated that AI-generated differentials were clinically relevant and could potentially lead to appropriate interventions, thereby aligning with patient safety and standards [34]. Additionally, evaluators ranked the match of differential to the final diagnoses. Conversely, a score of 0 was given if the differential diagnosis list significantly differed from the final diagnosis, indicating a lack of clinical relevance or potential misdirection in a real-world diagnostic scenario. Any discrepancies were resolved by another expert physician (KT), ensuring objective and consistent evaluation across all included case reports.

### Outcome

The study assessed the diagnostic accuracy of ChatGPT-4V, as an intervention and compared it to ChatGPT-4 without vision as a control. The primary outcome was defined as the ratio of cases where the final diagnoses were included within the top 10 differential diagnosis lists. The secondary outcome is defined as the ratio of cases where the final diagnoses were included as top diagnosis. These outcomes were chosen to quantitatively measure diagnostic accuracy and the effectiveness of image data integration in enhancing ChatGPT-4’s diagnostics.

Additionally, we assessed the contributing weight between text data (case descriptions) and image data (files) in developing the differential diagnosis lists, as reported by ChatGPT-4V. The total contribution from both elements was set to 100%. Specifically, we analyzed how much the text and image data individually contributed to the formulation of the differential diagnosis list. For example, if the text data (case description) contributed 60% and the image data contributed 40%, the total would sum up to 100%. This method allowed for a comprehensive understanding of the relative impact of textual and image data on AI diagnostics.

### Statistical Analysis

For analysis, R (version 4.2.2; R Foundation for Statistical Computing) was used. We present continuous variables as medians and IQRs to accurately reflect the distribution of data. We presented categorical or binary variables as numbers and percentages. Additionally, we used *χ*^2^ tests to compare categorical variables, setting the significance level at a *P* value <.05. The choice of *χ*^2^ tests for comparing categorical variables was based on their ability to handle binary and categorical data effectively, providing a robust measure of association between diagnostic outcomes and ChatGPT-4 with or without vision.

To quantify the impact of each factor on the likelihood of accurate diagnosis inclusion, an univariable logistic regression model was applied. This model allows for the exploration of potential predictors of diagnostic accuracy, offering insights into how different data types contribute to ChatGPT-4’s diagnostic processes. For the logistic regression model, the primary and secondary outcomes were treated as binary dependent variables: presence (1) or absence (0) of the correct diagnosis within the top 10 differential diagnosis lists and as the top diagnosis, respectively. Independent variables included the proportion of image data weight, the presence (1) or absence (0) of specific types of image data (eg, computed tomography [CT] images, pathological specimens, laboratory data, magnetic resonance imaging [MRI] scans, and X-ray images), and the number of characters in the text data. Odds ratios (ORs) and associated 95% CIs were used to estimate the relative risks of potential predictors of the final diagnosis included within the top 10 differential diagnosis lists in the univariable logistic regression model.

## Results

### Case Descriptions and Image Data Profile

A total of 363 case descriptions with additional image data, such as figures or tables, were included. ChatGPT-4V generated the differential diagnosis lists for all case descriptions. Representative final diagnosis, image data, and differential diagnosis lists generated by ChatGPT-4V and ChatGPT-4 without vision are shown in Table 2. The cases included in this study, along with the differential diagnosis lists generated by ChatGPT-4V and without vision, are shown in Multimedia Appendix 1.

**Table 2:** Representative final diagnoses, image data, and differential diagnosis lists generated by ChatGPT-4 with vision and ChatGPT-4 without vision.

Final diagnoses	ChatGPT-4 with vision (intervention)				ChatGPT-4 without vision (control)
	Image data	Image data weight^a^, %	Differential diagnosis lists	Differential diagnosis lists	
Olanzapine intoxication	Laboratory data	20%	Hospital-acquired pneumonia Sepsis Acute kidney injury Respiratory failure Intensive care unit delirium Hypotension Opioid intoxication Benzodiazepine intoxication Hyperglycemia Cardiovascular complications after surgery	Zolpidem overdose or prolonged effect Benzodiazepine overdose Olanzapine overdose or side effect Interaction between zolpidem and olanzapine Delirium Other drug overdose Severe sepsis Neuroleptic malignant syndrome Acute cholinergic crisis Acute stroke	
Secondary syphilis	Laboratory data and X-ray images	40%	Osteoarthritis Rheumatoid arthritis Gout Pseudogout Systemic lupus erythematosus Psoriatic arthritis Reactive arthritis Osteoporosis Septic arthritis Ankylosing spondylitis	Secondary syphilis Reactive arthritis Rheumatoid arthritis Osteoarthritis Gout/pseudogout Psoriatic arthritis Lyme disease Systemic lupus erythematosus Infectious arthritis Vasculitis (eg, granulomatosis with polyangiitis and microscopic polyangiitis)	
Sarcomatoid carcinoma	Computed tomography image and pathological specimens	30%	Colorectal cancer Adenocarcinoma of the colon Malignant mesothelioma Gastrointestinal stromal tumor Carcinoid tumor of the appendix Lymphoma Desmoid tumor Metastatic carcinoma Leiomyosarcoma Neuroendocrine tumor of the colon	Colorectal carcinoma Metastatic malignancy to the colon Peritoneal mesothelioma Sarcomatoid carcinoma Malignant mixed Mullerian tumor (carcinosarcoma) Gastrointestinal stromal tumor Leiomyosarcoma Colonic lymphoma Malignant peripheral nerve sheath tumors Undifferentiated/unclassified malignancies	

^a^The proportion of image data weight contributing to development of the differential-diagnosis lists.

Among these, the 25th percentile, median, and 75th percentile number of characters in the text data were 1971, 2683, and 3442, respectively. The maximum and minimum number of characters in text data were 7148 and 465, respectively. Regarding image data, CT images, pathological specimens, laboratory data, MRI scans, and X-ray images were included in 163, 124, 98, 77, and 70 case descriptions, respectively. The details of image data are shown in Multimedia Appendix 2.

### Diagnostic Performance

**Figure 2:**
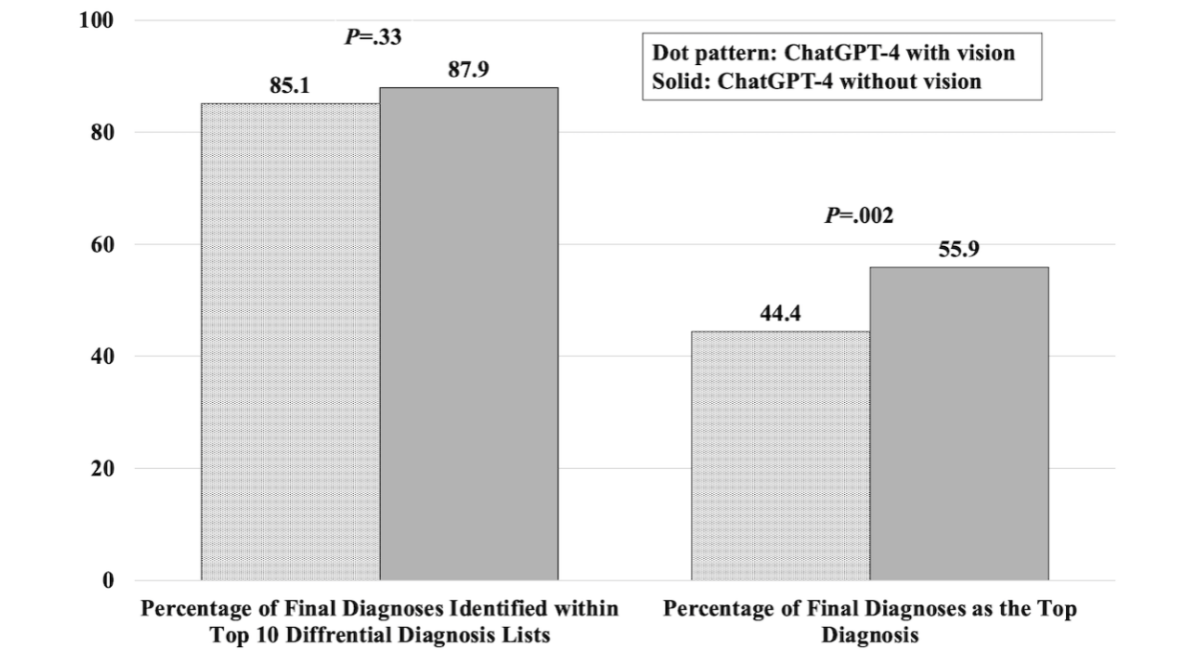
The rate of final diagnoses within the top 10 differential diagnosis lists and as the top diagnoses generated by ChatGPT-4 with vision and without vision.

For the primary outcome, the rate of final diagnoses within the top 10 differential diagnosis lists generated by ChatGPT-4V was 85.1% (n=363), compared with 87.9% (n=363) by ChatGPT-4 without vision (*P*=.33). For the secondary outcome, the rate of final diagnoses as the top diagnoses generated by ChatGPT-4V was 44.4% (n=363), inferior to 55.9% (n=363) by ChatGPT-4 without vision (*P*=.002). Figure 2 shows the rate of final diagnoses within the top 10 differential diagnosis lists and as the top diagnoses generated by ChatGPT-4V and without vision.

### The Contributing Weight Between Text and Image Data in Developing the Differential Diagnosis Lists

**Figure 3:**
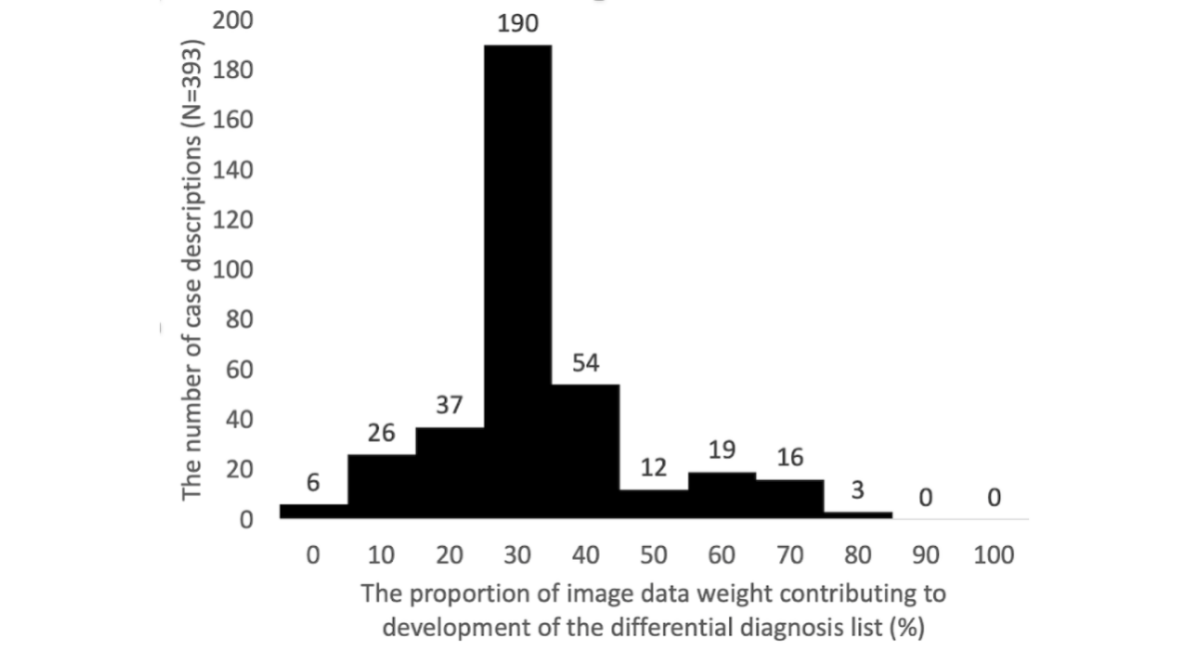
The proportion of image data weight contributing to the development of the differential diagnosis lists by ChatGPT-4 with vision.

The 25th percentile, median, and 75th percentile proportions of image data weight contributing to the development of the differential diagnosis lists were 30%, 30%, and 40%, respectively, indicating a consistent reliance on image data across a significant portion of cases. The maximum and minimum proportion of image data weight contributing to the development of the differential diagnosis lists were 80% and 0%, respectively, highlighting the wide range of reliance on image data across different case reports. Specifically, in 190 case descriptions of the total 363 included case reports (190/363, 52.3%), the proportion of image data weight contributing to the development of the lists was reported to be 30%. Figure 3 shows the proportion of image data weight contributing to the development of the differential diagnosis lists.

### The ORs of Variables for Predicting the Outcomes

**Figure 4:**
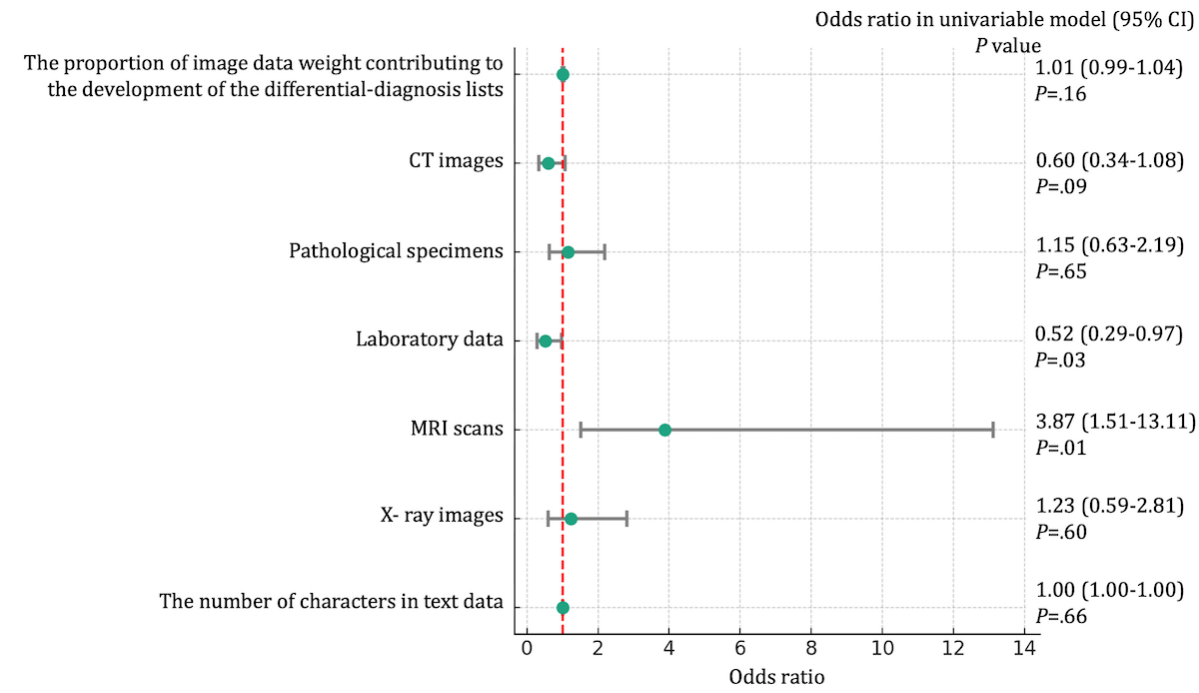
Odds ratios of variables for predicting the final diagnoses included within the top 10 differential diagnosis lists by ChatGPT-4 with vision in univariable regression model. *P* values are derived from the univariable logistic regression model. CT: computed tomography; MRI: magnetic resonance imaging.

Laboratory data independently predicted the inclusion of the final diagnoses within the top 10 differential diagnosis lists by ChatGPT-4V: OR 0.52 (95% CI 0.29-0.97; *P*=.03). Additionally, MRI scans were also found to be independent predictive factors: OR 3.87 (95% CI 1.51-13.11; *P*=.01). These results were derived from univariable logistic regression models. Other variables, including the proportion of image data weight contributing to the development of the differential diagnosis lists, CT images, pathological specimens, X-ray images, and the number of characters in text data, were not associated with the final diagnoses included within the top 10 differential diagnosis lists by ChatGPT-4V, as shown in Figure 4.

**Figure 5:**
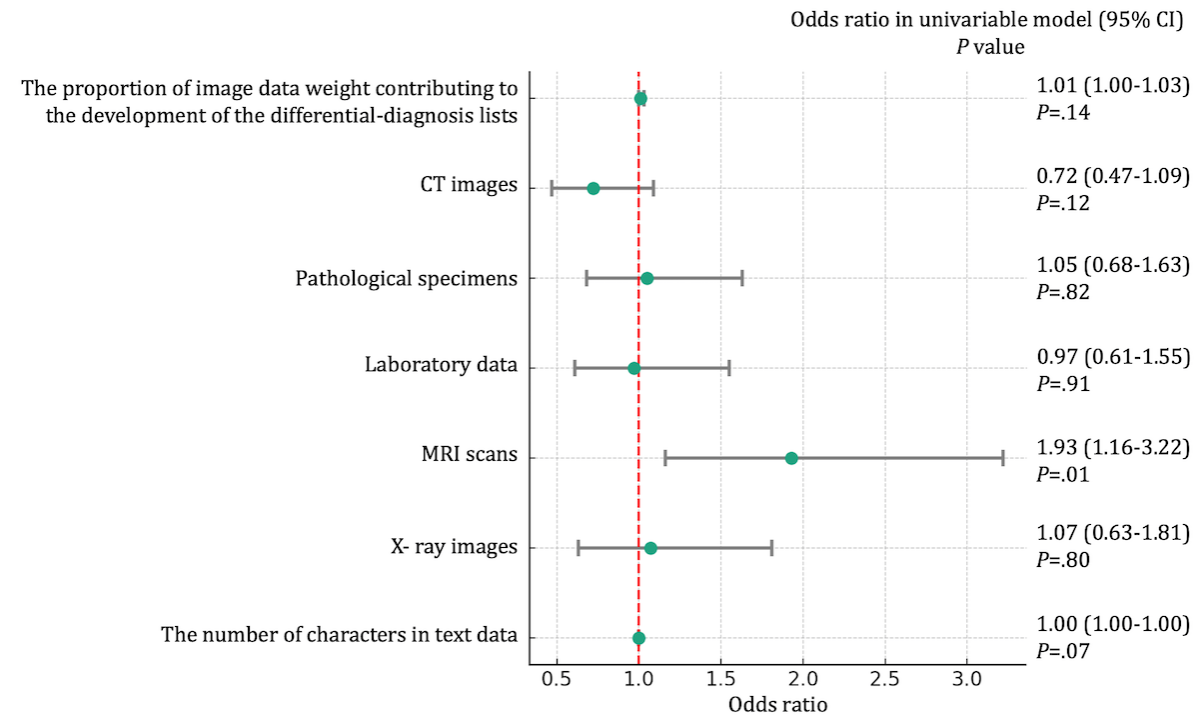
Odds ratios of variables for predicting the final diagnoses as top diagnoses by ChatGPT-4 with vision in univariable regression model. *P* values are derived from the univariable logistic regression model. CT: computed tomography; MRI: magnetic resonance imaging.

Additionally, MRI scans (OR 1.93, 95% CI 1.16-3.22; *P*=.01) were independent predictive factors for the final diagnoses as top diagnoses by ChatGPT-4V. Other variables were not associated with the secondary outcome, as shown in Figure 5.

## Discussion

### Principal Results

This study showed several key findings regarding the diagnostic capabilities of ChatGPT-4 with and without vision. The incorporation of image data into ChatGPT-4V did not yield a significant improvement in diagnostic accuracy compared with that without vision. This was evident in the rates of final diagnoses within the top 10 differential diagnosis lists generated by ChatGPT-4V, where ChatGPT-4 without vision actually demonstrated comparable performance. Conversely, the rate of final diagnoses as the top diagnoses generated by ChatGPT-4V was inferior to that without vision. While ChatGPT-4V accepts a wide range of medical images, from physical examinations to various investigation results, its potential to enhance diagnostic accuracy appears underused. This underuse of image processing capabilities could be attributed to the current AI model’s limitations in processing and integrating complex image data with textual data. Additionally, the AI system’s training regimen, which might have emphasized text data over image data, could have resulted in a bias toward text-based analysis. Future iterations of AI systems should focus on enhancing the model’s ability to discern and integrate key diagnostic features from both text and images.

In the univariable logistic regression model, these findings suggest that while the integration of image data by ChatGPT-4V did not uniformly improve diagnostic accuracy across all cases, specific types of image data, particularly MRI scans, play a crucial role in certain diagnostic contexts. MRI scans were associated with significantly higher rates of primary and secondary outcomes. Conversely, laboratory data were associated with significantly lower rates of the primary outcome. These results suggest that MRI scans are typically focused on specific body locations to target particular organs. For example, the inclusion of brain MRI scans led ChatGPT-4V to focus its differential diagnoses on cerebral diseases. The characteristics of MRI scans to focus on anatomical regions could be used to enhance the diagnostic performance of ChatGPT-4V in identifying specific conditions. Moreover, the laboratory data, often presented in tables, typically cover a broader spectrum of information than the case descriptions. For instance, in the case of infectious diseases with elevated blood glucose levels which were included only in the table, ChatGPT-4V considered hyperglycemic condition in addition to the final diagnoses. Incorporating additional laboratory data into the textual analysis could broaden the differential diagnosis lists, potentially reducing the primary outcome. The logistic regression analysis thus provides valuable insights into how different data formats influence the AI’s diagnostic capabilities, guiding future improvements in AI design and training to better leverage these inputs.

Focusing on the proportion of image data weight contributing to the development of the differential diagnosis lists, a notable observation emerges regarding ChatGPT-4V’s reliance. In more than half of the outputs, image data accounted for 30% of the weight in developing the differential diagnosis lists. This finding leads us to consider the system’s internal decision-making process. It is important to consider that the accuracy of the proportion of image data weight in representing the actual process of integrating text and image input in ChatGPT-4V remains uncertain. Despite the consideration, the proportion of image data weight further indicates a dominant dependence on text data. It raises the possibility that ChatGPT-4V may not be integrating text and image inputs in a balanced way. The implication here is that even with its capability to process image data, the system’s diagnostic output might still be mainly influenced by text data.

Given these findings, this unexpected outcome leads us to question why additional image data did not contribute to improvements in diagnostic accuracy. Exploring the reasons behind these results, one plausible explanation emerges related to the potential biases in ChatGPT-4V’s use of image data. The biases would be rooted in its training regimen. Rather than aiding in diagnosis, this image data could introduce complexity, leading ChatGPT-4V to rely more on text-based analysis and less on visual clues.

This study highlights the challenges in harnessing the full potential of multimodal AI in medical diagnostics. The findings indicate that despite the advanced capabilities of ChatGPT-4V, its integration of image data is not yet optimizing diagnostic outcomes. This would be partly because of the system’s inherent design and training, which could predispose it to prioritize text over image data, despite the latter’s potential richness in clinical information. This revelation is crucial for the ongoing development of AI in health care, highlighting a pivotal area for improvement. As AI continues to evolve, focusing on the harmonious integration of text and image data will be essential. This study paves the way for future innovations, guiding efforts to refine multimodal AI systems for more accurate, efficient, and reliable medical diagnostics. Future research should particularly explore the development of more sophisticated methods for image analysis and the optimization of multimodal data integration, aiming to improve the current reliance on text data and enhance the diagnostic power of AI in health care settings.

The findings from our study also raise important considerations for the practical application of AI in health care. While AI systems like ChatGPT-4V hold promise for supporting clinical decision-making, their current limitations necessitate a cautious approach to integration into clinical workflows. For instance, AI could serve as a supporting tool for preliminary analysis, helping triage or providing a second opinion in diagnostic challenges, thereby augmenting the expertise of health care professionals rather than replacing it. Health care professionals should be aware of these systems’ strengths and weaknesses, leveraging them as support tools rather than definitive diagnostic solutions.

### Limitations

There were several limitations in this study. A major limitation of our study was the reliance on selected image data excerpted from case reports [35], rather than whole slices of image data from clinical settings. This limitation partly arose because the current ChatGPT-4V can only process partial slices of image data [27]. This approach, while necessary for concise reporting in cases, may not accurately reflect the complexity and variability encountered in real-world clinical practice. Moreover, we excluded video files. Although generative AI systems currently do not accept video files, their inclusion could potentially improve diagnostic accuracy. Future research should explore incorporating more comprehensive image data sets and video data, technologies permitting, to enhance the AI system’s diagnostic capabilities. Furthermore, the study’s reliance on data derived from case reports may not encompass the diversity of real-world clinical scenarios [36]. The specificity of data sources inevitably impacts the generalizability of our findings, highlighting a significant challenge in extending our results to different health care settings and populations. Future studies should consider including complete data from real-patient scenarios with various situations.

Beyond these specific limitations, our study underscores broader concerns regarding the integration of AI in health care, particularly the potential bias inherent in the data sets used to train generative AI systems like ChatGPT-4. These biases may impact the generalizability of the AI’s diagnostic and predictive capabilities across diverse populations and clinical settings. The absence of regulatory approval for generative AI systems in clinical practice further complicates their potential adoption, while inconsistencies in ChatGPT-4V interpretations of medical imaging underscore the current limitations of these technologies in performing medical functions [25].

Furthermore, the interpretability and explainability of AI-generated diagnoses remain significant hurdles [16]. The deployment of AI in health care settings also raises practical challenges related to the training of health care professionals in AI use and the integration of AI tools into existing clinical workflows. Ensuring that health care workers are adequately prepared to interpret AI-generated insights and make informed decisions is crucial for the successful adoption of AI technologies.

Last, the rapid evolution of AI technology presents unique challenges, as advancements may quickly outpace the findings of our study. The pace at which AI technologies evolve means that our conclusions may become outdated as new capabilities emerge. This highlights the importance of ongoing research and adaptation in the field of AI and health care, ensuring that studies remain relevant and that AI tools are continually evaluated and updated to reflect the latest technological advancements.

### Comparison With Prior Work

Compared with a previous preliminary study for ChatGPT-4V, this study showed higher performance. The previous study assessed the proficiency of ChatGPT-4V for selected medical images from open-source libraries and repositories [27]. The study reported that only 21.7% (n=15) of cases were correctly interpreted with the correct advice. This inconsistency was partly because of the methodological differences between the 2 studies, particularly in terms of data set preparation and evaluation criteria. While the previous study mainly focused on a limited data set with simple prompts and evaluated the system’s interpretation and medical advice quality, our study introduced a more comprehensive data set with a rich clinical context. Additionally, we evaluated the diagnostic accuracy, rather than merely assessing interpretation and advice, thereby providing a deeper insight into the AI system’s utility in clinical decision-making.

Another study evaluated the performance of ChatGPT-4V for selected clinical cases from the website, including image data [26]. The study showed that ChatGPT-4V heavily relies on the patients’ medical history. This result was consistent with this study that additional image data did not improve the diagnostic accuracy. The result was also consistent with this study that approximately half of the outputs reported that the proportion of image data weight contributing to the development of the differential diagnosis lists was 30%.

A critical distinction between our study and previous works is our comparative analysis of ChatGPT-4 with and without vision capabilities. This unique approach allowed us to highlight the impact of image data on diagnostic accuracy, revealing that while ChatGPT-4’s vision component does not significantly enhance diagnostic accuracy, it does not detract from it either. This finding is crucial for understanding the role of integrated image data in AI-assisted diagnosis and highlights the potential of AI systems to support health care professionals by providing a comprehensive analysis that includes both text and image data.

### Conclusions

The rates of final diagnoses within the differential diagnosis lists generated by ChatGPT-4V did not show improvement over those generated without vision. The rate of final diagnoses as the top diagnosis generated by ChatGPT-4V was inferior to that without vision. Despite its multimodal data processing capabilities, ChatGPT-4V appears to prioritize text data, which may limit its effectiveness in medical diagnostic applications, as highlighted by its system card [25]. The implications of our study for the advancement of multimodal AI systems in health care are profound. It uncovers a pivotal aspect of AI development that requires attention: the nuanced integration and weighted analysis of diverse data types. To emulate the complex reasoning of medical professionals, AI systems must advance beyond simple data incorporation toward a sophisticated synthesis that enhances diagnostic accuracy. For future improvements, we recommend the following: enhanced clinical data fusion techniques; interpretability of AI decisions; and collaborative development efforts with AI developers and medical professionals. In clinical practice, more sophisticated multimodal AI systems have the potential to enhance in providing timely, contextually rich differential diagnoses, serving as educational aids for medical trainees, and enhancing patient care by supporting remote or underserved areas. Through these enhancements, AI tools can ultimately improve patient outcomes.

## Appendix

Multimedia Appendix 1 The included cases in this study and the differential diagnosis lists generated by ChatGPT-4 with vision and without vision.

Multimedia Appendix 2 The details of image data in this study.

## Abbreviations

AI: artificial intelligence
CDSS: clinical decision support system
ChatGPT-4V: ChatGPT-4 with vision
CT: computed tomography
LLM: large language model
MRI: magnetic resonance imaging
OR: odds ratio
