# Supplementary material for: Evaluating ChatGPT-4’s Diagnostic Accuracy: Impact of Visual Data Integration
Source: JMIR Med Inform. 2024 Apr 9;12:e55627. doi: 10.2196/55627 (PMC11040438; doi:10.2196/55627)
Supplement: Multimedia Appendix 2 [file medinform_v12i1e55627_app2.docx]

Table S1. Details of image data in the current study.

|  | The number of case descriptions |
| --- | --- |
|  | N = 363 |
|  |  |
| **CT image, n (%)** |  |
|  | 163 (44.9) |
| **Pathological specimen, n (%)** |  |
|  | 124 (34.2) |
| **Laboratory data, n (%)** |  |
|  | 98 (27.0) |
| **MRI, n (%)** |  |
|  | 77 (21.2) |
| **X-ray, n (%)** |  |
|  | 70 (19.3) |
| **Ultrasonography, n (%)** |  |
|  | 45 (12.4) |
| **Physical examination, n (%)** |  |
|  | 39 (10.7) |
| **Clinical Course, n (%)** |  |
|  | 26 (7.2) |
| **Electrocardiography, n (%)** |  |
|  | 21 (5.8) |
| **Endoscopy, n (%)** |  |
|  | 15 (4.1) |
| **Fundoscopy, n (%)** |  |
|  | 5 (1.4) |
| **Genomic test, n (%)** |  |
|  | 3 (0.8) |
| **Audiometry, n (%)** |  |
|  | 2 (0.6) |
| **Autopsy, n (%)** |  |
|  | 2 (0.6) |
| **Flowcytometry, n (%)** |  |
|  | 2 (0.6) |
